# Excitotoxin-induced caspase-3 activation and microtubule disintegration in axons is inhibited by taxol

**DOI:** 10.1186/2051-5960-1-59

**Published:** 2013-09-09

**Authors:** Anna Elizabeth King, Katherine Adriana Southam, Justin Dittmann, James Clement Vickers

**Affiliations:** 1 Wicking Dementia Research and Education Centre, University of Tasmania, Private Bag 23, Hobart, Tasmania 7000, Australia; 2 Menzies Research Institute, University of Tasmania, Hobart, Tasmania 7000, Australia

**Keywords:** Axon degeneration, Caspase, Excitotoxicity, Taxol, Microtubule

## Abstract

**Background:**

Axon degeneration, a key pathological event in many neurodegenerative diseases and injury, can be induced by somatodendritic excitotoxin exposure. It is currently unclear, however, whether excitotoxin-induced axon degeneration is mechanistically similar to Wallerian degeneration, which occurs following axon transection, but does not involve axonal caspase activation.

**Results:**

We have used mouse primary cortical neurons at 9 days *in vitro,* in a compartmented culture model that allows separation of the axon from the soma, to examine the pathological cascade of excitotoxin-induced axon degeneration. Excitotoxicity induced by chronic exposure to kainic acid, resulted in axonal fragmentation, which was associated with activation of caspase-3 in the axonal compartment. To examine the role of microtubules in these events, the microtubule-stabilizing agent, taxol, was added to either the axonal or somatodendritic compartment. Our results demonstrated that microtubule stabilization of axons resulted in a significant reduction in the number of fragmented axons following excitotoxin exposure. Interestingly, taxol exposure to either the somatodendritic or axonal compartment resulted in reduced caspase-3 activation in axons, suggesting that caspase activation is a downstream event of microtubule destabilization and involves signalling from the cell soma.

**Conclusion:**

These data suggest that excitotoxin-induced axon degeneration shows some mechanistic differences to Wallerian degeneration, and that microtubule stabilization may assist in protecting nerve cells from excitotoxic effects.

## Background

Axon pathology is a feature of many neurodegenerative diseases recently reviewed in [1] and following brain trauma [2]. The causes of axon pathology and degeneration in specific disease and injury conditions is currently unknown and may arise from a variety of insults. However, there is general consensus that axon degeneration can arise independently of cell death [3,4]. In this respect, several studies have shown that somal protection in disease models may fail to protect the axons from degeneration and this is a potential cause of lack of beneficial clinical outcomes for many therapeutic agents despite obvious rescue from neuronal loss [5,6]. Two causes of axonal degeneration have been well documented in the literature. The first is axon transection, which results in complete disconnection of the distal axon from the soma and proximal axon regions, and the second is developmental axon pruning, which occurs during establishment of neuronal circuitry [7] and in cell culture is often modelled by growth factor withdrawal in dorsal root ganglion (DRG) neurons [8].

It has been well established that, following axon transection, the distal axon segment undergoes a cascade of stereotypical degenerative alterations, termed Wallerian degeneration. Much of what we know about Wallerian degeneration comes from the discovery of the *Wlds* mouse, which undergoes delayed Wallerian degeneration following peripheral axon transection, reviewed in [9,10]. This mouse expresses an abnormal fusion protein resulting from the splicing of two mRNAS which encode Nmnat1 (an NAD+ salvage enzyme) and the N-terminal protein of Ube4b (an E4 ubiquitin ligase) [11]. Although Wlds is protective in models of axonal severing [9] and a number of other disease models [12-14], it fails to protect against all forms of axonal degeneration in disease models [15-17] and does not protect against axon degeneration that occurs in developmental axon pruning [18]. Recent studies have highlighted other differences between Wallerian degeneration and developmental axon pruning, despite both being characterized morphologically by axonal beading and fragmentation. Simon et al. [8] demonstrated differences in proteolytic cascades in these types of degeneration, with developmental pruning being associated with axonal caspase-3 activation, through the mitochondrial activation of caspase-9. Caspase-3 activation is not involved in Wallerian degeneration [3,8]. Differences have also been demonstrated in the spatial activation of kinase pathways in these two forms of axon degeneration [19].

Axon degeneration in neurological disease is rarely associated with direct axonal severing and it is, thus, pertinent to investigate other potential causes of axon degeneration. Determining the pathological cascade within the axon will aid in developing therapeutic agents that will preserve axon integrity and function. Our previous studies and those of others, have demonstrated that both somatodendritic and axonal insults can result in a pathological cascade of axon degeneration *in vivo* and *in vitro*[20-27]. Of potential relevance to a number of neurodegenerative conditions, we have shown that chronic somatodendritic excitotoxin exposure in cultured cortical neurons results in degeneration of the unexposed axon [27]. Aberrant excitatory activity, including excitotoxicity, has been implicated in a number of neurodegenerative conditions, including amyotrophic lateral sclerosis (ALS), Alzheimer’s disease (AD), multiple sclerosis (MS), stroke, epilepsy and CNS trauma [28-31] many of which are characterized by extensive axonal degeneration [32,33], suggesting that excitotoxic mechanisms may contribute to axonal pathology in these conditions.

Axon degeneration following excitotoxicity in cultured cortical neurons has been characterized morphologically by axonal beading and fragmentation, sharing similarities with Wallerian degeneration and axon pruning. Axon degeneration involves a breakdown of the neuronal cytoskeleton, including microtubule depolymerisation [20] and early loss of neurofilament proteins [21] prior to frank destruction/dissolution of microtubules [20,21]. Microtubules are highly dynamic molecules capable of depolymerization at the ends (dynamic instability) and subject to severing along their length. These properties allow rapid growth and disassembly, which is essential for their role in cell division, motility, morphology and transport. The clear organization of MTs throughout the cell, their large surface area, polar configuration, highly dynamic properties and role in transport makes them an ideal candidate for the spatial organization of signal transduction molecules within different parts of the cell [34]. The role that microtubule stability plays in axon degeneration and, in particular, whether microtubule breakdown is a downstream process resulting from activation of proteolytic cascades or has a more active role in the onset of degradation, is currently unclear.

Therefore, in this *in vitro* study using cultured mouse cortical neurons, we have investigated axon degeneration following excitotoxicity induced by kainic acid to determine mechanistic similarities to other forms of axon degeneration. Utilizing compartmented microfluidic chambers that allow spatial separation and manipulation of the soma and axons, we have investigated the role of microtubule destabilization in subsequent axonal and dendritic pathology in relation to excitotoxicity, in addition to the potential role of activation of apoptotic cascades within the axon. Our study indicates that kainic acid induced axon degeneration involves the activation of caspase-3 within the axon compartment and is inhibited by stabilization of the axonal microtubule network. Our data suggests that excitotoxin induced axon degeneration shares features of both Wallerian degeneration and developmental axon pruning.

## Methods

### Preparation of microfluidic chambers

**Figure 1:**
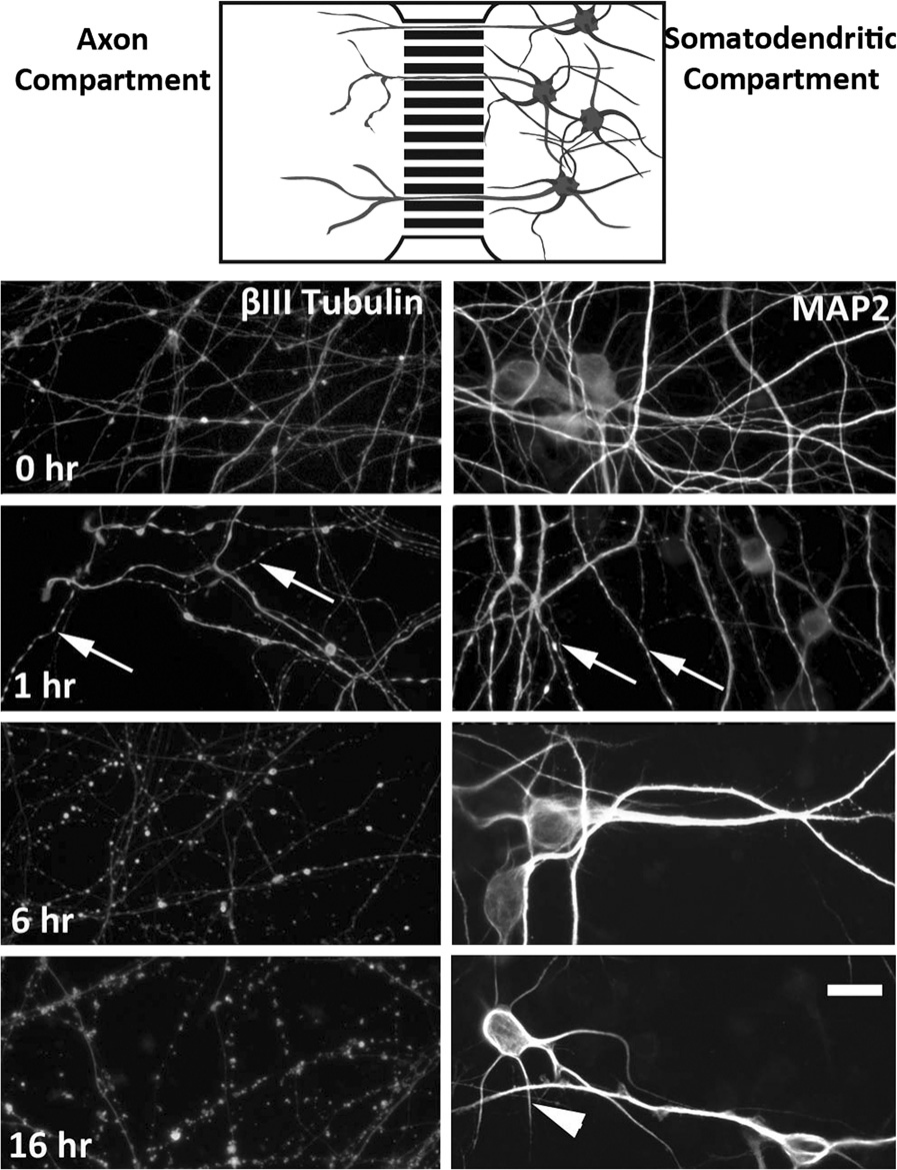
**Timecourse of axonal and dendritic alterations following somatodendritic exposure to kainic acid.** Top panel show schematic of neuronal arrangement in microfluidic chambers. Β-III tubulin (axonal) and MAP2 (dendrite) immunoreactivity indicated beading of a small number of both distal axons and distal dendrites by 1 hour post exposure to kainic acid. By 6 hours extensive axonal beading was present and dendritic arbours were reduced in area. By 16 hours axons were fragmented, although β-III tubulin immunoreactivity was still present within axonal fragments and MAP2 immunoreactivity was restricted to neuronal soma and a small number of short dendrites. Arrows indicate beading; arrow head indicates shortened dendrites. Scale bar: 20 μm.

Microfluidic chamber (Figure 1) were prepared as described previously [27]. Briefly, chambers were sterilized in ethanol, dried and attached to 22 mm^2^ glass coverslips (Marienfeld). Poly-L lysine substrate (0.001%, Gibco) was loaded into both sides of the chamber and left overnight. Substrate was removed and chambers were filled with cell plating media consisting of Neurobasal media (Gibco), 2% B27 supplement (Gibco), 0.5 mM glutamine, 25 μM glutamate, 10% fetal calf serum (Gibco) and 1% antibiotic/antimycotic (Gibco). Chambers were equilibrated for a minimum of 4 hours prior to culture.

### Primary cell culture

Primary mouse cortical cell culture was performed as we have described previously [27,35]. Briefly, cortical tissue was dissected from E15.5 mouse embryos and dissociated using 0.0125% trypsin (5 minutes at 37°C). Tissue was washed in cell plating media followed by mechanical tituration using a 1 ml pipette. Cell viability and density was assessed using a trypan blue exclusion assay. Cell concentration was adjusted to 8×10^6^ cells/ml. Media was removed from the wells of the microfluidic chambers and 20 μl cells were loaded into one side of the microfluidic chambers (somatodendritic chamber). Cells were allowed to adhere to the substrate for 15 minutes prior to filling chambers with cell plating media. The following day, media was changed to cell maintenance media consisting of plating media without the fetal calf serum and glutamate. Cells were grown in 5% CO_2_ at 37°C.

### Pharmacological manipulation

Cells were treated with 100 μM kainic acid or vehicle control at 9 days *in vitro* (DIV) corresponding to a time of dense axonal growth in the axon chamber. For taxol treatments, 3 μg/ml taxol (in a maximum of 0.1% DMSO) or DMSO vehicle control (0.1%) was added to the axonal or somatodendritic chamber at the time of treatment with kainic acid.

### Immunohistochemistry

Cell cultures were fixed in 4% paraformaldehyde prior to immunolabelling. Primary antibodies including activated caspase-3 (Millipore, 1:200), β-III tubulin (Promega, 1:1000) and MAP2 (Millipore, 1:1000) were diluted in 0.3% triton-X (Fluka) and applied for 1 hour at room temperature and overnight at 4°C. Isotype and species specific secondary antibodies were applied at 1:1000 for 2 hours at room temperature. The nuclear stain DAPI was applied at for 15 minutes at room temperature.

### Live cell imaging and quantitation

#### *Taxol toxicity*

To determine taxol toxicity, propidium iodide was applied to live cultures for 5 minutes prior to imaging on a Leica (DM LB2) fluorescent upright microscope fitted with a 40× dipping lens. Propidium iodide labelling in the nucleus (indicating loss of cell viability) was quantitated in 100 neuronal cells (identified by morphological characteristics under light microscopy) in randomly selected fields of view.

#### *Axon fragmentation*

Live images of axons were acquired on a Nikon TiE motorized inverted microscope, with chambers maintained at 37°C. For quantitation of axonal degeneration, images were acquired of the axonal compartment prior to cell treatments and from identical areas at 16 hours after treatments. A 3X3 square grid was placed over images (total area 0.2 mm^2^) and the percentage of fragmented or intact axons was quantitated by an observer blinded to conditions. Numbers of fragmented axons were normalized to pre-treatment counts for individual images giving a reliable quantitation of change in axonal fragmentation. Three images for each coverslip were taken for quantitation and a minimum of n = total of 5 coverslips from 3 separate culture days were used for all analysis except the dose response data where n = total of 3 coverslips across 3 separate cultures.

#### *Dendritic arbour size*

To determine dendritic arbour length, treated and fixed chambers (n = total of 4 coverslips across 3 separate cultures) were immunolabelled with MAP2 and labelled with the nuclear stain DAPI. Six images were acquired from randomly selected fields of the somatodendritic compartment on a Leica (DM LB2) fluorescent upright microscope with a 40× objective. Acquisition parameters were constant between data sets. Dendritic arbourization was quantitated in ImageJ using the NeuriteTracer plugin using standard protocols, with thresholds constant between data sets for individual experiments.

#### *Caspase quantitation*

Caspase quantitation was performed on n = 5 coverslips from 3 separate cultures. Treated and fixed cultures were immunolabelled with an anti-activated caspase-3 antibody and 6 images were acquired from randomly selected fields of view of the axon compartment on a Leica (DM LB2) fluorescent upright microscope with a 40× objective. Positive labelling was selected form background using the threshold function of ImageJ with values constant between data sets. Area of positive immunoreactivity, as a proportion of total area was quantitated using ImageJ.

#### *Statistical analysis*

All statistical analysis and graphs were prepared in graphpad Prism (6.0) using one-way Anova with Bonferroni Post-hoc tests. P < 0.05 was considered significant.

## Results

### Somatodendritic kainic acid exposure induced axonal and dendritic cytoskeletal alterations

We have previously described morphological changes to axons following somatodendritic glutamate exposure [27]. To determine the sequence of cytoskeletal changes in the axons of mouse cortical neurons exposed to kainic acid in the somatodendritic compartment, treated (100 μM kainic acid) and untreated cultures were fixed over a timecourse of 1, 6 and 16 hrs and labelled with antibodies against neuron-specific β-III tubulin or the dendrite specific microtubule associated protein MAP2. Loss of MAP2 immunoreactivity in dendrites has been shown previously to be an early feature of excitotoxicity and other insults [35]. In untreated cultures, β-III tubulin was present throughout the axons with occasional blebbing in the axon (Figure 1). At 1 hour post exposure to kainic acid, there was a distinct beading of the distal terminals of many axons in the axon chambers (Figure 1). MAP2 immunoreactivity demonstrated that beading was also present in distal dendritic arbours (Figure 1). At 6 hours following kainic acid exposure, β-III tubulin immunoreactivity demonstrated increased beading throughout the axon. MAP 2 immunolabelling demonstrated loss of immunoreactivity in dendritic arbours at this stage relative to untreated cultures (Figure 1). By 16 hours, extensive axon fragmentation had occurred and β-III tubulin was highly beaded and fragmented, but still present in axonal fragments (Figure 1). MAP2 immunoreactivity in dendritic arbours following 16 hours kainic acid exposure was restricted to the soma and short lengths of dendrite (Figure 1).

### Kainic acid induced axon fragmentation involved destabilization of axonal microtubules, which was rescued by axonal taxol exposure

**Figure 2:**
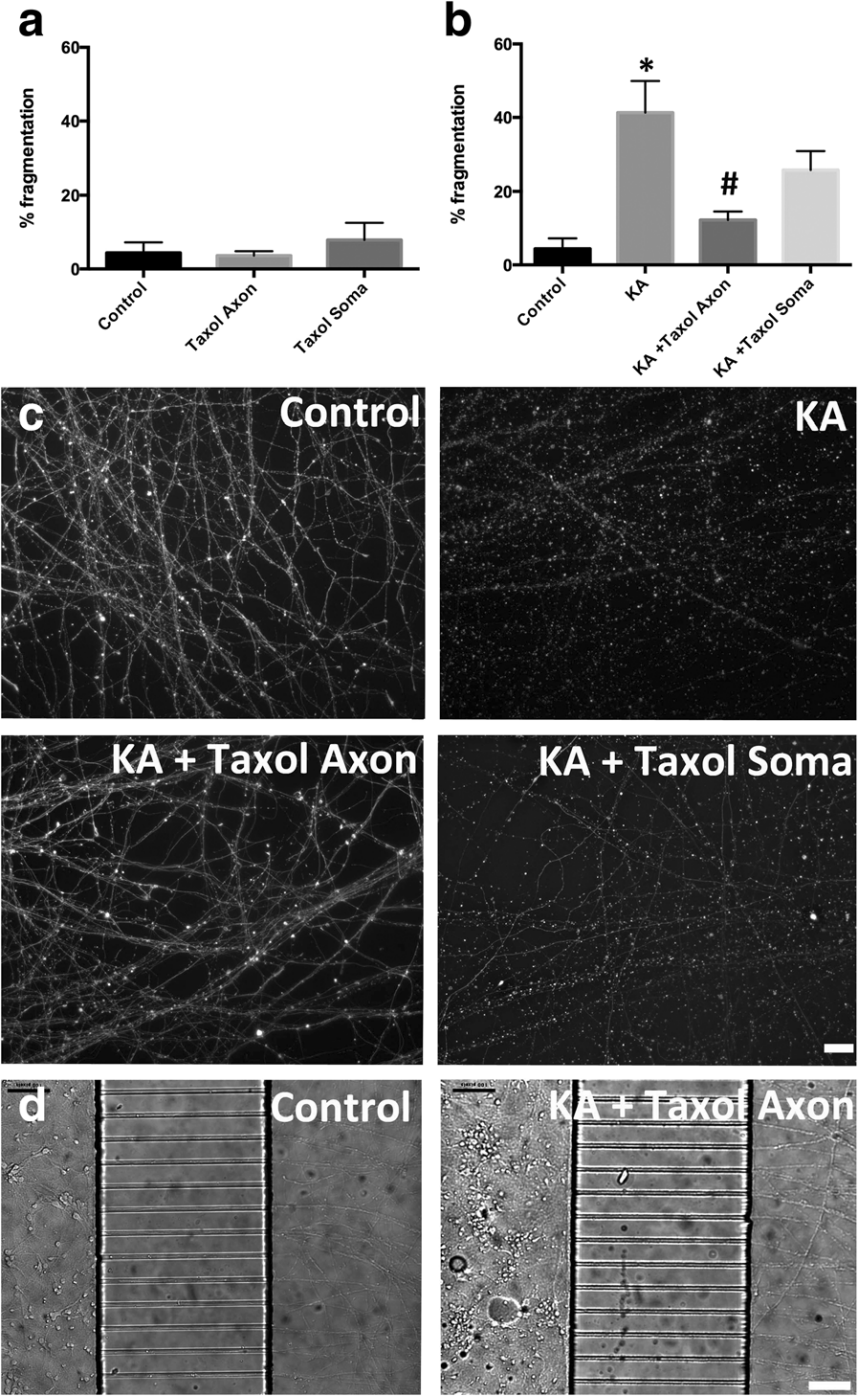
**Taxol rescue of kainic acid induced axon degeneration. a.** Taxol applied to axon or somatodendritic compartment alone did not induce axonal fragmentation. **b.** Kainic acid applied to the somatodendritic compartment induced a significant increase in axonal fragmentation in the axon compartment. Axonal, but not somatodendritic, taxol significantly protected the axons from kainic acid induced axon fragmentation. **c.** βIII tubulin immunoreactivity in the axon compartment demonstrates extensive fragmentation following kainic acid treatment relative to vehicle treatment (control). Axonal taxol treatment rescued the axons from fragmentation. **d.** Phase contrast image of control (vehicle) treated chamber shows healthy soma and unfragmented axons. Following kainic acid exposure, cell soma appear bright and condensed indicating degeneration, however taxol in the axon compartment maintains the integrity of the axons. KA, kainic acid. * Significantly different from control. # Significantly different from kainic acid treatment. P < 0.05. Mean values shown ± SEM. Scale bars: c, 20 μm; d, 100 μm.

**Figure 3:**
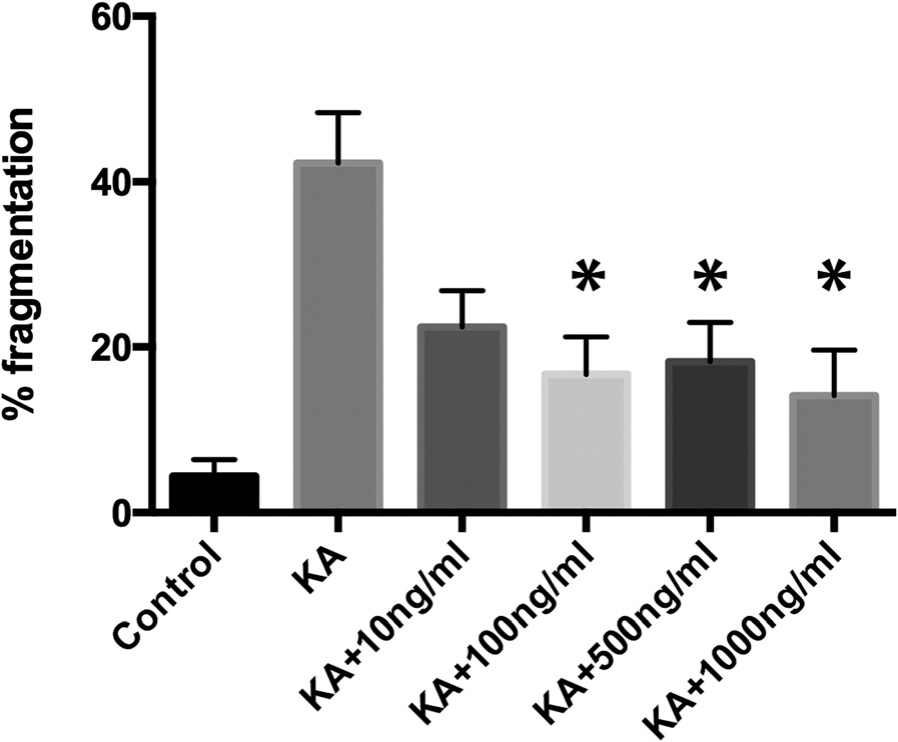
**Dose response curve of rescue of axonal fragmentation by taxol.** Graph shows axonal fragmentation in vehicle (control) treated cultures and those exposed to kainic acid (KA) with 10, 100, 500 and 1000 ng/ml taxol. 100 ng/ml taxol was sufficient to significantly protect axons from fragmentation following kainic acid exposure.

Our data showed that axon fragmentation and microtubule alterations are an early feature of excitotoxin induced axon degeneration. We next determined if preventing microtubule destabilization with the drug taxol, would rescue the axon from fragmentation. Initial investigations demonstrated that taxol concentrations of 1-3 μg/ml did not cause neuronal death over a period of 24 hours. However concentration of 10 μg/ml taxol caused some loss of nuclear membrane integrity, demonstrated by uptake of propidium iodide, indicating cell death (data not shown). For subsequent experiments, 3 μg/ml taxol or vehicle control was added to the somatodendritic or axonal compartment at the time of addition of kainic acid or vehicle to the somatodendritic compartment. Axon degeneration (fragmentation) was quantitated at 16-18 hrs post kainic acid treatment. Axonal or somatodendritic application of taxol alone did not cause a significant increase in axonal fragmentation in these cultures (Figure 2a). Kainic acid induced a significant (p < 0.05) increase in axon fragmentation (41.3 ± 8.6%, mean ± SEM) relative to vehicle-treated cultures (4.4 ± 2.9%, mean ± SEM, Figure 2b). Application of taxol to the somatodendric compartment did not significantly alter the percentage of fragmented axons (25.8 ± 5.2%, mean ± SEM, Figure 2b) induced by kainic acid, although there was a trend towards reduction. However, axonal exposure to taxol resulted in a significant protection of the axon with reduced axonal fragmentation (12.2 ± 2.3%, mean ± SEM) relative to kainic acid treated axons treated with vehicle alone (Figure 2b). These data indicate that taxol protects the axon directly by preventing microtubule destabilization within this compartment and that somatodendritic microtubule stabilization does not significantly prevent axon degeneration. To determine the concentration range that taxol is able to provide axonal protection against fragmentation, kainic acid treated cultures were exposed to axonal taxol from 10-1000 ng/ml. Axonal fragmentation was significantly reduced with 100 ng/ml taxol, while 10 ng/ml showed a non-significant trend towards reduced fragmentation (Figure 3).

### Stabilization of microtubules in the somatodendritic or axonal compartment does not prevent loss of dendritic MAP2

**Figure 4:**
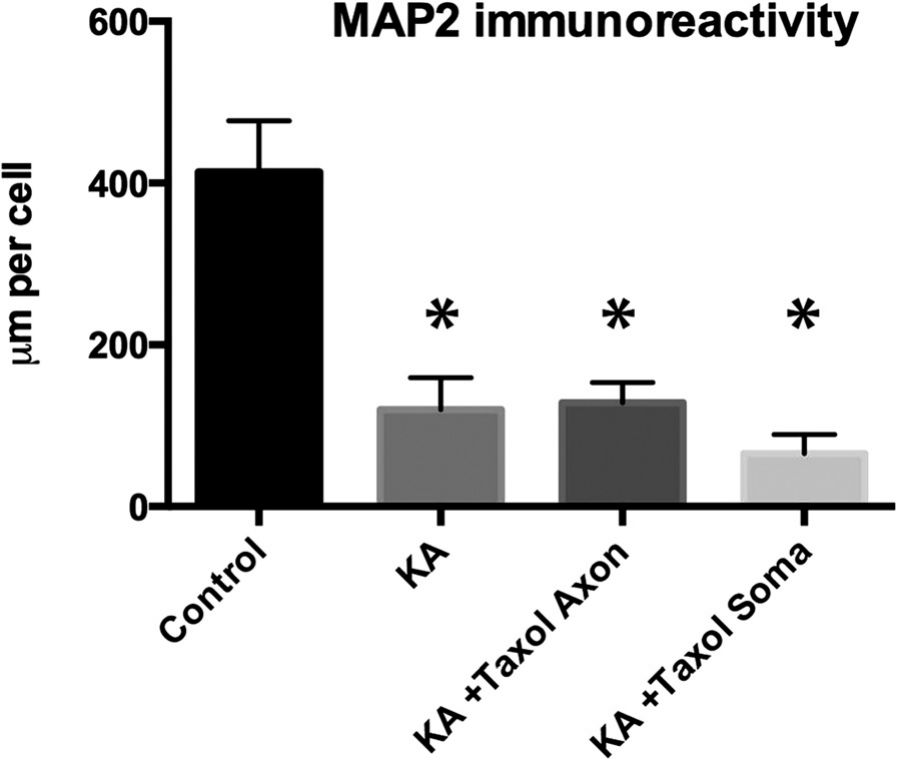
**Effect of kainic acid on dendritic arbour.** Kainic acid significantly reduced the length of MAP2 immunoreactive dendritic arbours and this was not prevented by taxol in either the axonal or dendritic compartment. KA, kainic acid. * Significantly different from control. P < 0.05. Mean values shown ± SEM.

Similar to axonal microtubule destabilization, alterations to MAP2 are an early feature of excitotoxin-induced axon degeneration. We next examined whether taxol protection in either the axonal or somatodendritic compartment prevented loss of MAP2 immunoreactivity in dendrites following kainic acid exposure. Application of taxol to either the axonal or somatodendritic compartment did not protect the neuron from loss of MAP2 immunoreactivity following kainic acid exposure (Figure 4).

### Kainic acid induced axon degeneration involved activation of caspase-3 in the unexposed axonal segment

**Figure 5:**
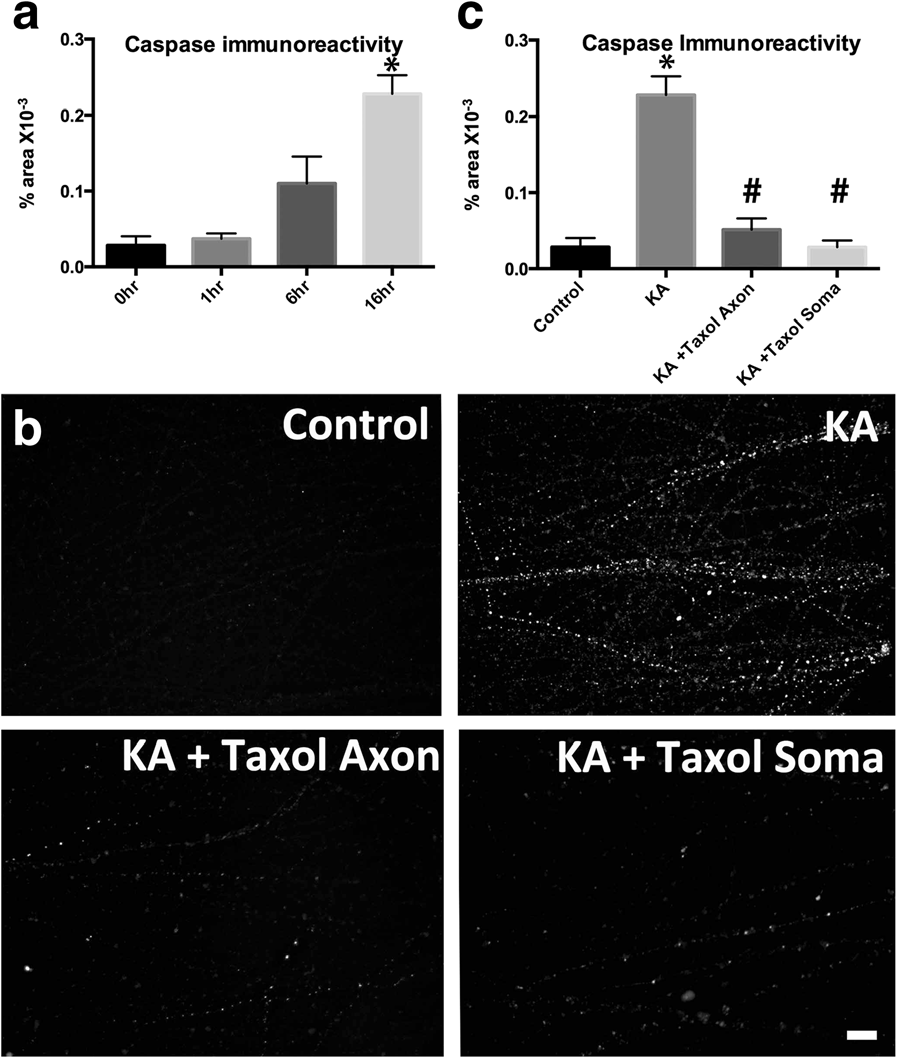
**Excitotoxicity induces activation of caspase 3 which is prevented by microtubule stabilization. a.** Active caspase-3 immunoreactivity was low in control (vehicle) treated chambers but was present in many axons as discrete puncta following kainic acid. Caspase immunoreactivity was not altered at 1 hour following kainic acid exposure but was significantly increased at 16 hrs. **b,c.** Kainic acid induced active caspase-3 immunoreactivity in the axon compartment was significantly attenuated by either somatodendritic or axonal taxol treatment. KA, kainic acid. * Significantly different from control. # Significantly different from kainic acid treatment. P < 0.05. Mean values shown ± SEM. Scale bar: 20 μm.

Cytoskeletal degradation can involve activation of proteolytic cascades. We investigated if axonal caspase activation was involved in axon degeneration following kainic acid exposure. Immunocytochemical analysis with an antibody against activated caspase-3 demonstrated that exposure to kainic acid resulted in induction of active caspase-3 in axons at 16–18 hours post treatment (Figure 5a,b). Immunoreactivity was present as regularly spaced puncta along the length of the axon and was present in axons in both the axonal and somatodendritic chambers. Caspase immunoreactivity was not present in MAP2 immunoreactive dendrites. To determine the staging of activation of caspase-3 following kainic acid exposure, cultures were treated and fixed over a timecourse of 1 and 6 hours. Increased numbers of caspase immunoreactive axons were not present at 1 hour post kainic acid exposure (Figure 5a). At 6 hours following kainic acid exposure there was a non-significant trend towards increase in caspase activation, which was variable between cultures, indicating that caspase activation occurs at a later stage of the degenerative process.

### Microtubule destabilization following kainic acid exposure is upstream of caspase activation

We next determined if axonal caspase activation occured prior to, or resulted from, microtubule destabilization. Kainic acid-treated cultures were co-incubated with taxol in the axonal or somatodendritic compartments (as described above) and fixed after 16–18 hours. Immunolabelling with active caspase-3 demonstrated that axonal taxol prevented kainic acid induced activation of caspase-3 in the axonal compartment with a significant reduction in caspase immunofluorescence in taxol-treated cultures (51.3 ± 14.7 × 10^-3^% of total area, mean ± SEM) relative to cultures exposed to kainic acid in the absence of axonal taxol (227.9 ± 24.5 × 10^-3^% of total area, mean ± SEM Figure 5b,c). This suggests that that caspase 3 activation occurs downstream of microtubule destabilization in the axon compartment. Interestingly, somatodendritic taxol application also significantly reduced activation of caspase-3 by kainic acid (28.5 ± 8.8 ×10^-3^% of total area, mean ± SEM), (Figure 5b,c) suggesting that somatodendritic signalling is involved in the activation of caspase in the axon.

## Discussion

Our previous studies and those of others have demonstrated that excitotoxicity can induce axon degeneration. However, the relationship between excitotoxin-induced axon degeneration and other forms of axon degeneration remain unclear. In this study we have investigated whether excitotoxiciy induces activation of axonal caspases as this has been implicated in developmental axon pruning, but is not involved in Wallerian degeneration following axon severing. Our study demonstrated that excitotoxicity results in activation of caspase-3 within the axon, suggesting a mechanistic difference to Wallerian-type degeneration. To investigate the relationship between cytoskeletal breakdown and caspase activation, we exposed cultures to excitotoxins in the presence or absence of the microtubule stabilizing agent, taxol, in the axonal or somatodendritic compartment, and examined the effect on axon fragmentation, axonal caspase activation and loss of dendritic MAP2. Our data demonstrated that application of axonal, but not somatodendritic, taxol, prevented axonal fragmentation. Furthermore both axonal and somatodendritic taxol prevented the activation of axonal caspase-3, although MAP2 loss was not altered. These data imply that caspase activation occurs downstream of microtubule destabilization within the axon and involves somal signalling.

### Axonal caspase 3 activation in axon degeneration

Caspases are cysteine proteases that are important in mediating apoptosis. However, recently caspases have been implicated in other cellular processes that are not associated with cell death including synaptic [36] and axon [8,37,38] pruning. Caspase activation in the axon has been associated with a number of neurodegenerative diseases and condition. For example, axonal caspase has been associated with beta amyloid toxicity [37] and is found following injury [39]. Furthermore in models of Krabbes disease, which is caused by deficiency in galactosylsphingosine and characterized by demyelination, caspase activation in the axon, but not the soma, is present prior to demyelination [40]. These data suggest that axonal caspase activation in these conditions represents a type of axon degeneration that is different from classical Wallerian degeneration.

Despite these finding the axonal role of caspases remain unclear. Caspases have a number of substrates, including cytoskeletal proteins, which could account for their activation. Both precaspase-3 and biochemically active caspase-3 have been shown to be present in healthy axons of cultured neurons [38] although the epitope of active caspase-3 may be masked by binding to apoptosis inhibitors as it can not be detected by immunohistochemistry. However, active caspase-3 immunoreactivity was localized to tubulin aggregates following nerve growth factor withdrawal [38], suggesting release from their inhibitors during degeneration. This is consistent with our findings of punctate expression of active caspase-3 following excitotoxicity. Growth factor deprivation may be involved in axonal degeneration following excitotoxicity as excitotoxicity has been shown to disrupt axon transport mechanisms and can result in the disruption and cleavage of components of the dynein- dynactin complex [41]. These molecular motors are responsible for retrograde transport and signaling, thus specifically implicating reduced growth factor signaling from the axon terminal [41].

Caspase activation following growth factor withdrawal is eliminated by knockdown of Bax [37,38], although pan caspase inhibitors do not provide complete protection against axon degeneration [38]. In this respect, caspase activation may act in parallel with other axon degeneration pathways involving NAD+ in this model [38]. This is similar to our previous findings in which cortical neurons treated with glutamate underwent axon degeneration that was partially protected by pan caspase inhibitors [27]. This suggests that caspase activation is not the only axon degeneration mechanisms activated in these pathways and also fits with our current study demonstrating that preventing axonal caspase activation by application of somatodendritic taxol does not prevent axon fragmentation. Thus, although the role of caspase-3 activation in axon degeneration is unclear, it may not be critical to degeneration and alterations to microtubule proteins may be more important in axonal maintenance.

### The role of microtubule destabilization in excitotoxicin induced axon degeneration

Microtubule fragmentation has previously been implicated in axonal degeneration mechanisms and was the earliest detectable change in axons following severing when examining cytoskeletal alterations [20]. Breakdown of the microtubule network in Wallerian degeneration involves the ubiquitin proteasome system. However, while this is an early event relative to cytoskeletal breakdown, the role of microtubule dynamics in the initial intracellular biochemical cascade that results in the breakdown of the cytosol, remain unclear. An early study demonstrated the protective role of microtubule stabilization with taxol on hippocampal neurons exposed to kainic acid, [42]. Similarly the microtubule stabilizing agent davunetide protects against kainic acid excitotoxicity in hippocampal cells both *in vitro* and *in vivo*[43]. However in these studies taxol was globally applied to neurons and protection attributed to prevention of calcium influx through calcium permeable AMPA receptors [42]. The role of microtubules in the axonal compartment was not examined. Interestingly in our study, somatodendritic taxol did not significantly protect the unexposed axon compartment, although a trend to reduced fragmentation was observed. This may reflect differences in the neurons cultured or suggest that axonal taxol was also contributing to axonal protection in the previous study.

Recently protective effects of axonal taxol have been reported in neurons exposed to 3 hours glutamate, strengthening our current results and suggesting that microtubule destabilization is an early and upstream event in axon degeneration following excitotoxicity [41]. Similarly several studies have now demonstrated protective effects of microtubule stabilization or increased microtubule acetylation in other models involving axonal degeneration such as mutant tau induced axon degeneration [44-46] and models of ALS [47]. In this respect microtubule stabilization protects neurons from activation of cdk5 and subsequent tau hyperphosphorylation in a cell culture model of amyloid toxicity [48] again suggesting that microtubule destabilization is an upstream event. Similarly, studies looking at dendritic degeneration following NMDA insult demonstrate that microtubule stabilization is upstream of calpain activation and loss of MAP2, suggesting that loss of MAP2 is not the cause of microtubule destabilization in this instance [49]. However in our study somatodendritic microtubule stabilization did not prevent MAP2 loss following kainic acid exposure perhaps reflecting difference in the excitotoxic pathways activated or the length of exposure.

Although microtubule stabilization is protective against degeneration in a number of models, overstabilization of microtubules may also be detrimental as seen in cases of peripheral neuropathy in cancer patients treated therapeutically with taxol [50]. Furthermore mutations in small heatshock proteins in Charcot Marie tooth disease cause them to bind more strongly to microtubules and alterations in microtubule dynamics may be the cause of peripheral neuropathy in this disease. Thus precise balance in microtubule stability may be necessary to prevent axonal degeneration mechanisms.

### Somal- axonal signaling in axon degeneration

The effect of compartment specific microtubule stabilization is of interest in the current study. We show that stabilization of axonal microtubules significantly reduced caspase-3 activation within the axon suggesting that microtubule destabilization is required for caspase activation. However, the effect of somatodendritic taxol on axonal caspase activation suggests that axonal microtubule destabilization alone is not sufficient for caspase activation, but that signalling from the soma to axon may be involved. The nature of this signal is currently unclear, however a recent study by Chen and colleagues [19] demonstrated that following growth factor withdrawal, spatially distinct kinase pathways are activated that differentially affect axonal beading and degeneration. In particular GSK3 activation or transcriptional repression in the soma was sufficient to block axonal degeneration but not axonal beading, whereas ErbB and p38 inhibition in the axon prevented both beading and fragmentation [19]. These data suggest that axon and soma may regulate different aspects of axonal degeneration and that in our excitotoxicity model these may be regulated in part by microtubule dynamics. This strengthens the hypothesis that parallel degeneration pathways are involved in excitotoxin induced axon degeneration. Despite this, although an early event, microtubule stabilization is not sufficient to prevent cleavage of components of the dynein-dynactin complex [41].

## Conclusion

The current study suggests that excitotoxin induced axon degeneration involves features of both Wallerian degeneration and growth factor withdrawal and that alteration in microtubule dynamics are an upstream event in axon degeneration. Further deciphering the alterations to microtubule dynamics in relation to their post translational modifications, binding of microtubule associated proteins and association with downstream signalling pathways will be important in providing effective therapeutic intervention in a number of degenerative diseases and conditions in the central nervous system.

## Competing interest

The authors declare that they have no conflict of interest for this manuscript.

## Authors’ contributions

AK designed and performed the experiments, analysed data and drafted the manuscript. KS performed analysis on images from cultures. JD prepared microfluidic cultures. JV aided in study design and manuscript preparation. All authors read and approved the final manuscript.
